# The prevalence of eHealth literacy and its relationship with perceived health status and psychological distress during Covid-19: a cross-sectional study of older adults in Blekinge, Sweden

**DOI:** 10.1186/s12877-022-03723-y

**Published:** 2023-01-04

**Authors:** Sarah Nauman Ghazi, Jessica Berner, Peter Anderberg, Johan Sanmartin Berglund

**Affiliations:** 1 Department of Health, Blekinge Institute of Technology, SE-371 79 Karlskrona, Sweden; 2 School of Health Sciences, University of Skövde, SE-541 28 Skövde, Sweden

**Keywords:** eHealth literacy, COVID-19, Psychological distress, Health status, Gerontology, Aging and care, Public health, eHealth

## Abstract

**Background and aims:**

eHealth literacy is important as it influences health-promoting behaviors and health. The ability to use eHealth resources is essential to maintaining health, especially during COVID-19 when both physical and psychological health were affected. This study aimed to assess the prevalence of eHealth literacy and its association with psychological distress and perceived health status among older adults in Blekinge, Sweden. Furthermore, this study aimed to assess if perceived health status influences the association between eHealth literacy and psychological distress.

**Methods:**

This cross-sectional study (October 2021-December 2021) included 678 older adults’ as participants of the Swedish National Study on Aging and Care, Blekinge (SNAC-B). These participants were sent questionnaires about their use of Information and Communications Technology (ICT) during the COVID-19 pandemic. In this study, we conducted the statistical analysis using the Kruskal-Wallis one-way analysis of variance, Kendall’s tau-b rank correlation, and multiple linear regression.

**Results:**

We found that 68.4% of the participants had moderate to high levels of eHealth literacy in the population. Being female, age $< 75$ years, and having a higher education are associated with high eHealth literacy ($p< 0.05$). eHealth literacy is significantly correlated ($\tau$=0.12, *p*-value=0.002) and associated with perceived health status ($\beta$=0.39, *p*-value=0.008). It is also significantly correlated ($\tau$=-0.12, *p*-value=0.001) and associated with psychological distress ($\beta$=-0.14, *p*-value=0.002). The interaction of eHealth literacy and good perceived health status reduced psychological distress ($\beta$=-0.30, *p*-value=0.002).

**Conclusions:**

In our cross-sectional study, we found that the point prevalence of eHealth literacy among older adults living in Blekinge, Sweden is moderate to high, which is a positive finding. However, there are still differences among older adults based on factors such as being female, younger than 75 years, highly educated, in good health, and without psychological distress. The results indicated that psychological distress could be mitigated during the pandemic by increasing eHealth literacy and maintaining good health status.

## Introduction and Background

The importance of digital tools in maintaining health and the capability required to use these commodities have been an important topic of discussion during COVID-19 [1]. Physical and psychological distress challenges have been evident during this pandemic [2, 3]. The mimicking symptoms with the common flue [4], the misinformation and rumors [5], news and harmful social media content [6] have caused much stress and panic in the world. Whereas, lack of social engagement, hospital visits, and general checkups due to restrictions in COVID-19 has led to an increase in the use of the internet for social purposes and seeking health information [7]. In this scenario, there is a need for the masses to have adequate knowledge about how and where to seek the correct information using the internet, evaluate, understand, and apply this information correctly, i.e., to have eHealth literacy.

eHealth literacy (eHL) has been defined by Norman and Skinner [8] as an “an individual’s ability to seek, understand and appraise health information from electronic resources and make informed health decisions for addressing a health problem in everyday activities”. This ability can help a person filter the misinformation and correctly understand and apply the correct information, which otherwise might cause stress and anxiety related to COVID-19 [9]. The pandemic has also prompted an increase in the use of eHealth resources to help in coping with the psychological repercussions [10].

eHealth literacy impacts how the individuals search health information on the internet, which influences health outcomes [11]. It is positively associated with lifestyle behaviors and health [12]. It is related to better behavioral and cognitive outcomes [13]. It entitles people with skills to make the right health-related decisions. Inadequate eHealth literacy may conceivably lead to poor health behaviors, thus impacting health [14].

Older adults might not be able to utilize the benefits of eHealth resources as they tend to use the internet and digital tools less often than younger ones [15]. They may even lack the skills and knowledge to use these resources to maintain health [16]. With the advancement of technology, eHealth literacy requires continuous improvement, especially for older adults [17]. Having an adequate eHealth literacy will improve older adults’ ability to manage their chronic conditions and minimize the negative effects on their health [18].

Relationship with eHealth literacy and psychological health has been studied in China [19] and Turkey [20]. These studies looked at multiple good age groups and the main focus was not on older adults explicitly. Studies also show a negative correlation between sorrow and anxiety and eHealth literacy among low-income older adults [21].

As for psychological distress, it refers to general symptoms of stress, anxiety and depression [22]. Moreover, one of the main psycho-social problems during the COVID-19, loneliness [23], co-occurs with psychological distress [24]. With the continuous development of eHealth resources, it is important to know if the ability to use eHealth resources can help in mitigating psychological distress. Moreover, very little attention has been paid to the effect of eHealth literacy on perceived health status and psychological distress during COVID-19. Besides this, the combined effect of the eHealth literacy and health status on psychological distress has also not been clarified before. Further, previous research has mainly focused on adolescents and adults, who are considered to use the internet more frequently for health-related purposes[25].

Therefore, in addition to analyzing the prevalence of eHealth literacy and its association with psychological distress (anxiety/depression and loneliness), this study aims to investigate if eHealth literacy and health status as perceived co-jointly affect psychological distress.

### Aim

The primary aim of this study is to analyze the prevalence of eHealth literacy in older adults living in Blekinge, Sweden, and assess the association of eHealth literacy, psychological distress and perceived health status. Our secondary aim is to measure eHealth literacy’s interactive effect (combined effect) and perceived health status on psychological distress.

## Method

### Study design and Participants

In this study, a cross-sectional survey was conducted under the longitudinal research project ‘Swedish National study on Aging and Care’ (SNAC) [26]. The study population contained all the participants above 65 years who are part of the SNAC study in Blekinge except those with severe cognitive disorder and those too frail to answer the questions. Out of 678 participants from the study population, 188 were non-respondents and 490 participants were included in our final sample for analysis.

### Data collection

**Fig. 1:**
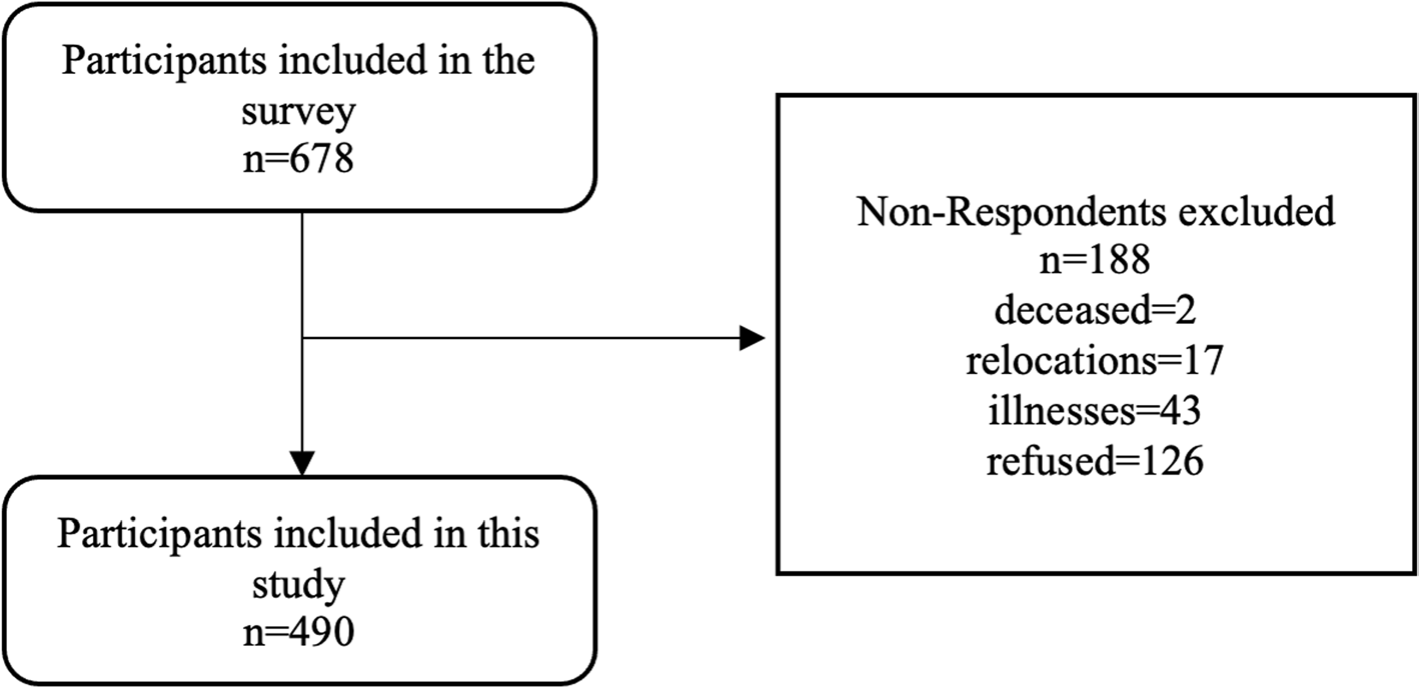
Sample flowchart

Questionnaires were sent to 678 participants aged 65 and above either through mail or email according to their preference. Participants were given the opportunity to not respond to the questionnaire on the main page, as well as information on the nature of the questions. Instructions on how to return the completed questionnaire, and the contact information for seeking help in case of any clarifications needed was also provided with the questionnaire. The participants were also reminded if they did not respond at first by sending the questionnaire for the second time. The data was collected from October 2021 to December 2021. A response rate of 72.2% was achieved ($n=490$). The non-respondents included 2 deceased persons, 17 relocations, and 43 illnesses that prevented participation; the remaining 126 did not return the questionnaire. Figure 1 shows the sample diagram.

### Measures

Age, gender, education, economic status, and living arrangement are the socio-demographic variables included. Age was used as a continuous variable for the central tendency of the sample and regression and categorized into three groups (65-74, 75-84, 85+ years). Gender was defined by Male and Female. The Swedish old education system relevant to our participants’ age is categorized into three groups: low, middle, and high levels of education. The economic status was assessed through a non-invasive question and categorized into good and poor. The participants were asked if they could get 17000 Swedish Krona (SEK) in one week if an unpredicted expense arrived. The participants economic status were categorized as good or poor based on this question regarding financial means. The participants were also asked about their living arrangement, whether they lived alone or with someone. They were also asked if they or their family or friends were infected with COVID-19.

The internet users were asked about eHealth literacy through an instrument developed by Norman and Skinner called ‘eHeals’. This instrument is an 8-item, 5-point Likert scale (1=Strongly disagree to 5=Strongly agree) that measures participants’ perceived skills in finding, evaluating, and utilizing eHealth information to keep themselves healthy [27]. This scale has been validated for the Swedish population in the study by Wanked et al. [28]. In our study, the total score(8-40) was presented as a mean score(1-5) to represent the weighted average of each item in the total score. This was calculated by dividing each participant’s total score with the number of items in eHeals. eHeals score i.e the eHealth literacy was also categorized into four groups  [29], Lack of eHealth literacy (8-15.99), low (16-23.99), moderate (24-31.99) and high eHealth literacy (32-40). In this study, the scale has a Cronbach’s Alpha reliability coefficient of 0.96, making it an instrument with high internal consistency.

A 5-point Likert-type item evaluated about perceived health status. The question asked was, “How is your health in general”. The Likert scale item ranged from 1 being ‘Excellent’ to 5 being ‘Bad’. It was categorized as ‘0’ Poor and ‘1’ as ‘Good’ perceived health status.

Psychological distress was measured with a General Health Questionnaire-12 (GHQ-12) sub-scale (Anxiety/depression) [30]. The sub-scale of Anxiety/depression has been used previously in a study to assess mental distress [31]. The participants were also asked about loneliness. The formulated question was ‘how often do you feel lonely’, and the responses were three items: Almost never, sometimes, and often. These items from these scales (Anxiety/depression, loneliness) were combined into a single variable ‘psychological distress’ through factor analysis. Each item of the scale was multiplied by its factor loading and then summed to constitute a score. The Cronbach’s alpha of these items after factor analysis was 0.83.

### Statistical analysis

The data was analyzed using STATA version:16.1 (developed by STATACorp LLC, College Station, TX, USA). Shapiro-Wilk test determined the normality of the non-likert scale variables. Descriptive statistics were used to describe all the variables, while central tendency (mean and standard deviation) of all the continuous variables were used to describe the center of the data distribution. Missing data constituted less than 5% of the sample, hence it was omitted. For prevalence of eHealth literacy, the descriptive statistics and Kruskal-Wallis one-way analysis of variance was performed. Kendall’s Tau-b rank correlation and multiple linear regression were used to investigate the association of eHealth literacy, psychological distress and perceived health status. In both multiple regression Models 1 and 2, the dependent variable was eHealth literacy, and the independent variables were perceived health status and psychological distress respectively. Model 3 had psychological distress as a dependent variable and the interaction of perceived health status and eHealth literacy as an independent variable. Included as control variables or covariates are age, gender, education, living conditions, economic status, and whether themselves or their family or friends have been infected with COVID-19.

### Ethical consideration

All the SNAC-B participants signed an informed consent after being recruited with the purpose to inform and acquire permission to use their responses in this research project. The participants were reassured that their anonymity and dignity would be respected, and they had the right to withdraw anytime. The Research Ethics committee of Lund University (LU 604-00) approved this study.

## Results

### Descriptive analysis

The sample characteristics are shown in Table 1. The mean age in the sample was 78 years (SD: 2.4, Range: 66 -101). Out of the total sample, 53.4% were female and 42.27% had secondary level of education. Most of the participants had good economic status (94.02), and live with someone (64.29%). 76.7% of the participants are internet users. Only 2.37% of the participants were infected with COVID-19 while 50.34% had family or friends that were infected with the virus. The participants’ perceived health status ranged from 1-5 with 2.93 as mean (SD:0.95). The mean of eHealth literacy was 3.44 (S.D: 1.26; Range: 1-5). Psychological distress in the population was 5.62 (S.D: 1.95; Range: 3.8-13.7)

**Table 1:** Socio-demographic characteristics of the participants ($N=490$) where ‘n’ is the number of participants. Central tendency of continuous variables are expressed as mean, standard deviation and range

Variables	n (%)	Mean (SD)(Range)
**Gender**		
Male	228 (46.53)	
Female	262 (53.47)	
**Age (years)**		77.9 (7.49) (66-101)
Less than 75	222 (45.31)	
75-84	143 (29.18)	
85+	125 (25.51)	
**Living conditions**		
Alone	171 (35.19)	
Not Alone	315 (64.81)	
**Education**		
Elementary	82 (23.91)	
Secondary	145 (42.27)	
Higher	116 (33.82)	
**Economic status**		
Good	330 (94.02)	
Poor	21 (5.98)	
**Internet users**		
User	376 (76.73)	
Non User	114 (23.27)	
**Infected with COVID-19**		
Yes	11 (2.37)	
No	453 (97.63)	
Total	464	
**Infected family or friends with COVID-19**		
Yes	224 (50.34)	
No	221 (49.66)	
Total	445	
**Perceived health status**		
Bad	22 (4.63)	
Fair	141 (29.68)	
Good	182 (38.32)	
Very Good	105 (22.11)	
Excellent	25 (5.26)	
Total	475	
**eHealth literacy**	364	3.44(1.266) (1-5)
**Psychological distress in COVID-19**	464	5.62 (1.95)(3.82-13.69)

### eHealth literacy in the population and its distribution

The levels of eHealth literacy and their distribution among internet users across the gender and age groups are shown in Table 2. Median score of eHealth literacy in the sample population was 3.69.

**Table 2:** Distribution of the four levels of eHealth Literacy among Gender and Age groups

		AGE GROUPS					
eHealth literacy	n (%)	Less Than75 n (%)		75-84 n (%)		85+ n (%)	
		Male	Female	Male	Female	Male	Female
Lack of literacy	54 (14.84)	7 (7.78)	11 (10)	10 (18.18)	5 (10.42)	11 (31.43)	10 (38.46)
Low literacy	61(16.76)	19 (21.11)	9 (8.18)	10 (18.18)	12 (25.0)	5 (14.29)	6 (23.08)
Moderate literacy	86 (23.63)	26 (28.89)	23 (20.91)	17 (30.91)	11 (22.92)	7 (20.00)	2 (7.69)
High literacy	163 (44.78)	38 (42.22)	67 (60.91)	18 (32.73)	20 (41.67)	12 (34.29)	8 (30.77)
**Total**	364 (100)	90 (100)	110 (100)	55 (100)	48 (100)	35 (100)	26 (100)

The prevalence of moderate to high eHealth literacy is 68.41% (249/364). Table 3 shows the central tendency (median) among different groups and significance value of the difference of eHealth literacy among various socio-demographic characteristics. Kruskal-Wallis test showed statistically significant difference of eHealth literacy among gender ($\tilde{\chi }^2$=5.53, *p*=0.018), age groups ($\tilde{\chi }^2$=17.5, *p*=0.000), education ($\tilde{\chi }^2$=8.16, *p*=0.01) and perceived health status ($\tilde{\chi }^2$=25.26 *p*=0.0001).

**Table 3:** Central tendency (median) of eHealth Literacy and Kruskal-Wallis test to determine statistically significant differences of eHealth Literacy between the groups

Variables	eHealth literacy Median	*p*-value
**Gender**		
Male	3.38	0.018$^{*}$
Female	4	
**Age (years)**		
Less than 75	4	0.0002$^{*}$
75-84	3.38	
85+	2.88	
**Living conditions**		
Alone	3.5	0.22
Not Alone	3.75	
**Education**		
Elementary	3	0.016$^{*}$
Secondary	3.63	
Higher	4	
**Economic status**		
Good	3.75	0.903
Poor	3.88	
**Infected with COVID-19**		
Yes	4.19	0.07
No	3.75	
Total		
**Infected family or friends with COVID-19**		
Yes	3.75	0.71
No	3.75	
Total		
**Perceived health status**		
Bad	4.63	
Fair	2.88	0.0001$^{*}$
Good	3.63	
Very Good	3.94	
Excellent	4.5	

$$^{*}$$ Statistically significant

**Fig. 2:**
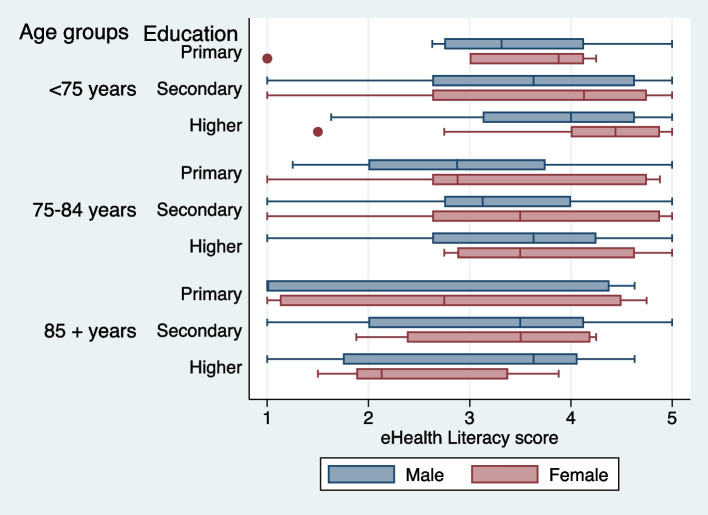
Box plots of eHealth literacy in older adults in Blekinge according to, gender, age group and education

Our results also showed that among males, there is no significant difference of eHealth literacy in all three age groups ($\tilde{\chi }^2$= 4.89, *p*=0.08) while among females we found a significant difference of eHealth literacy in all three age groups ($\tilde{\chi }^2$= 11.94, $p=0.002$). This can also be visualized in boxplot Fig. 2.

### Kendall’s Tau-b rank correlation

Kendall’s Tau-b rank correlation between eHealth literacy, perceived health status and psychological distress showed significant correlation ($< 0.05$). There was a negative significant correlation between eHealth literacy and psychological distress as well as perceived health status and psychological distress. The results of this correlation are presented in Table 4.

**Table 4:** Kendall’s tau ($\tau$)correlation between eHealth literacy (eHL), perceived health status (PHS) and Psychological distress (PD)

	eHL	PHS	PD
eHL	1		
PHS	($\tau$) = 0.1217$^{*}$ *p*-value: 0.0029 $^{*}$	1	
PD	($\tau$) = -0.1212 *p*-value: 0.0017 $^{*}$	($\tau$) = -0.2346$^{*}$ *p*-value: $< 0.0001^{*}$	1

$$^{*}$$ Statistically significant

### Multiple linear regression

We projected three regression models where we kept the socio-demographic variables (age, gender, education, economic status, living condition, infected with COVID-19, and infected family or friends with COVID-19) constant in every model. Table 5 shows all the models. For every model we also conducted regression analysis without the control variables to check if control variables impact the independent variable, which is shown in Table 6.

**Table 5:** Multiple regression: Association of eHealth literacy (eHL), perceived health status (PHS) and psychological distress (PD) in Sweden older adults with Socio-demographic variables as control or covariates. Dependent variables for Model 1 and 2 is eHL and for model 3 it is PD. Independent variable for Model 1 is PHS, Model 2 is PD and Model 3 in interaction of eHL and PHS

Independent Variables	Model 1 (eHL)		Model 2 (eHL)		Model 3 (PD)	
	$\beta$	*p*-value	$\beta$	*p*-value	$\beta$	*p*-value
**Age**	-0.03	0.007$^{*}$	-0.04	0.001$^{*}$	-0.02	0.337
**Gender Women**	0.32	0.043$^{*}$	0.41	0.01$^{*}$	0.75	0.001$^{*}$
**Education Secondary Higher**	0.20 0.43	0.406 0.076	0.08 0.31	0.74 0.19	-0.69 -0.59	0.033 0.072
**Living condition**	0.02	0.89	-0.10	0.57	-0.73	0.002$^{*}$
**Economic status**	0.19	0.40	0.36	031	1.04	0.035$^{*}$
**Infected with COVID-19**	0.54	0.27	0.54	0.27	0.03	0.834
**Infected family or friends with COVID-19**	0.09	0.54	0.12	0.45	0.17	0.435
**Perceived Health status**						
*Poor*	Ref.					
*Good*	0.33	0.07				
**Psychological distress**			-0.14	0.002$^{*}$		
**eHL x PHS**						
*Poor PHS*					-0.01	0.34
*Good PHS*					-0.30	0.002$^{*}$
**R2**	0.16		0.14		0.19	
**Adjusted R2**	0.07		0.11		0.16	
***p-value***	0.001		0.0001		$< 0.0001$	

$^{*}$ Statistically significant, eHL= eHealth Literacy, PD= Psychological Distress, PHS= Perceived Health Status

Model 1 with eHealth literacy as dependent variable and perceived health status as independent. It did not show significance ($\beta$=0.33, *p*-value= 0.07). However, when the control variables were not considered, the results showed significant positive association ($\beta$=0.39, *p*-value= 0.008$^{*}$).

In model 2 eHealth literacy was negatively associated with psychological distress ($\beta$= -0.14, *p*-value= 0.002).

Model 3 predicted the interactive effect of eHealth literacy and perceived health status on psychological distress. Poor perceived health status results were insignificant ($\beta$= -0.01, *p*-value= 0.34) but significant negative association was found between the interaction of eHealth literacy and good perceived health status ($\beta$= -0.30, *p*-value= 0.002).

**Table 6:** Multiple regression: Association of eHealth literacy (eHL), perceived health status (PHS) and psychological distress (PD) in Sweden older adults without control or covariates. Dependent variables for Model 1 and 2 is eHL and for model 3 it is PD. Independent variable for Model 1 is PHS, Model 2 is PD and Model 3 in interaction of eHL and PHS

Independent Variables	Model 1 (eHL)		Model 2 (eHL)		Model 3 (PD)	
	$\beta$	*p*-value	$\beta$	*p*-value	$\beta$	*p*-value
**Perceived Health status**						
*Poor*	Ref.					
*Good*	0.39	0.008$^{*}$				
**Psychological distress**			-0.10	0.005$^{*}$		
**eHL x PHS**						
*Poor PHS*					-0.04	0.68
*Good PHS*					-0.24	0.002$^{*}$
**R2**	0.02		0.02		0.05	
**Adjusted R2**	0.01		0.01		0.04	
***p-value***	0.007		0.004		$< 0.001$	

$^{*}$ Statistically significant, eHL= eHealth Literacy, PD= Psychological Distress, PHS= Perceived Health Status

## Discussion

Considering the lack of studies on eHealth literacy (eHL) with psychological distress (PD), and perceived health status (PHS) during COVID-19 in older adults, we aimed to examine the level of eHealth literacy among older adults residing in Blekinge, Sweden, and assess its association with psychological distress, and perceived health status. A secondary aim was to evaluate the combined impact of increasing eHealth literacy and good perceived health status on psychological distress.

The key findings of this study are:

- Most (68.4%) of the participants have moderate to high levels of eHealth literacy in Blekinge, Sweden. Females, less than 75 years of age and higher education among older adults are associated with good eHealth literacy.
- The relationship between perceived health status and eHealth literacy, especially during COVID-19, is influenced by socio-demographic factors.
- Psychological distress is significantly associated with reduced eHealth literacy.
- Higher eHealth literacy and good health status have a cumulative effect, beneficial for the older adults with PD.

The findings of this study are especially relevant to the delivery of relevant eHealth care services to older adults [32]. Our study indicated that 14.8% of the older adults lack eHealth literacy despite using the internet. As internet use declines with age among older adults [15], the results showed that eHealth literacy also declines with age. There are several possible explanations for this result. It can be attributed to the older adults’ attitudes toward the Internet, which include computer anxiety, computer self-efficiency, computer confidence, or preference for in-person interaction with the health practitioner [33, 34]. eHealth literacy may also be lower due to age-related problems such as vision and hearing problems, cognitive and health decline [35]. It is of interest to overcome these barriers with assistive technologies for example, in order to increase eHealth literacy and help older adults improve their health [11, 18].

Further, results from this study showed that females have a higher eHealth literacy level than males. Generally, women have a more positive attitude towards the use of internet and online resources for health information than men [36]. They also use health-related content on websites significantly more frequently than men [37]. The socio-demographic information regarding eHealth literacy will help identify the need assessment for the target population when implementing interventions.

Although our results show that women have higher eHealth literacy than men, in general, it can be seen in the distribution (Fig. 2) that females highly educated tend to have lower eHealth literacy in the oldest old age group (85+ years) compared to males in the same category. Women who have highly demanding jobs are often more educated and more likely to use their cognitive reserve, which may lead to stronger cognitive decline [38]. According to one study, the oldest old women have a negative perception of aging than middle aged women which is strongly associated with less internet use [39], which may be another explanation for why women above 85+ have a low eHealth literacy. This might be the possible explanation that instead of having a higher education, in our result women above 85 years of age show a low eHealth literacy in our study. Other explanations for the unequal eHealth literacy between gender, age and education is a topic worth probing in future studies. This study also pointed out that higher education is associated with higher eHealth literacy which although consistent with its association with internet use [15] and previous studies [40, 41], but it is not always a better predictor of eHealth literacy [42]. It is important to take into consideration the bias of low education and age when promoting eHealth services or interventions in order to ensure that those in need of these services are not left out [43].

Findings from this study also showed that eHealth literacy is correlated with health status and its association is influenced by other socio-demographic factors. The good health status of older adults will allow them to use information communication technology (ICT) for health effectively [41] and thus better eHealth literacy. Increasing eHealth literacy is also associated with better behavioral and cognitive outcomes [13]. In addition to our study, other studies have also indicated the association between eHealth literacy and perceived health status [12, 14]. eHealth literacy also affects health-promoting behaviors in general as seen during the pandemic [12, 44] thus improving health [13]. eHealth literacy is also helpful for the adequate management of chronic conditions like diabetes which can consequently improve the health condition  [45].

This study also found that older adults with increasing psychological distress exhibited reduced eHealth literacy. A previous study states that sorrow and anxiety do not inhibit internet use in older adults, but it does negatively correlate with eHealth literacy [21]. Increasing psychological distress might hinder eHealth literacy. Studies suggest that psychological health affect the cognitive function which among several things include learning ability [46, 47]. This could explain the association of psychological distress and eHealth literacy found in our result [19, 48]. Another association found in several studies is that of psychological distress and perceived health status [49, 50]. Our study went on to show that better-perceived health and increased eHealth literacy would help to reduce psychological distress. This suggests that we can decrease psychological distress in older adults by improving their eHealth literacy and focusing on their health status.

Overall, our study filled the knowledge gap of the prevalence of eHealth literacy among older adults in Blekinge, Sweden, and the sociodemographic factors that are of influence. It also adds to the knowledge that psychological distress during COVID-19 can be managed by improving the eHealth literacy and older adults’ self-perception of health. Our findings may help in the development of appropriate interventions aimed at older adults who are in the most need.

### Strengths and Limitations of the study

One of the main strengths of this study is that of the sample. The sample of older adults from SNAC closely reflects that of the general Swedish population. It is randomly selected and highly representative of this longitudinal study. It is a privilege to have this kind of data, even if we did not use it for longitudinal purpose. Another strength of this study is the use of validated instruments. Cronbach’s alpha of all the instruments was above 0.8 indicating high internal validity. Although the external validity is not so robust, the samples nevertheless provided useful information that can be seen as a future reference for interventions concerning eHealth literacy, Psychological distress during COVID-19 in Sweden. Another limitation is that this study was in the context of the pandemic when loneliness and anxiety were on the rise. A different time-setting study would be helpful to get a complete picture. Thirdly, the study population is only from Blekinge, which is a small to mid-size town. Perhaps investigating rural urban Sweden with a larger sample would give us different health results of the older adults.

Furthermore, the nature of this study is that it is cross-sectional. Therefore, one can not establish the causality of the variables discussed in this study. Finally, this study is confined to those who use the internet, which suggests that a focus on how people acquire health information may be necessary to promote eHealth.

## Conclusion

It is of great importance to understand the impact of eHealth literacy on perceived health status and psychological distress, specifically during the current pandemic. It can help older adults to manage and maintain their health. The findings from this study show that eHealth literacy is unequally distributed among the older adults with low educated men of age 75+ years have insufficient eHealth literacy. Good eHealth literacy and good perceived health status have a combined negative effect on psychological distress. This evidence suggests that improving eHealth literacy and the health status of older adults might be a way to mitigate psychological distress during COVID-19.

## Data Availability

The datasets used and/or analyzed during the current study are available from the SNAC-Blekinge principal investigator (Johan Sanmartin Berglund) on reasonable request.
